# Segmental dynamics and local motions in disordered random copolymers

**DOI:** 10.1021/acs.macromol.6c00032

**Published:** 2026-04-18

**Authors:** Stavros X. Drakopoulos, Sundol Kim, Richard A. Register, Rodney D. Priestley

**Affiliations:** a Princeton Materials Institute, 6740 Princeton University, Princeton 08540, New Jersey, United States; b Department of Chemical and Biological Engineering, 6740 Princeton University, Princeton 08540, New Jersey, United States

## Abstract

Understanding the glass transition in amorphous polymers and the underlying principles that govern segmental motions remains a key challenge in polymer physics. Here, we investigated random copolymers composed of methyl methacrylate (MMA) and 4-*tert*-butylstyrene (TBS) monomers across various compositions to elucidate the influence of monomer bulkiness on the glass transition temperature (*T*_g_), fragility (*m*), and segmental dynamics. The calorimetric *T*_g_ values were observed to strongly deviate from the Fox equation, showcasing a structure-independent behavior at high to medium MMA concentrations, and a ‘super-Fox’ increase at low MMA content. We correlated this to tacticity as well as *frustrated* chain packing, as corroborated by changes in the *m* values. A closer look at the β-relaxation revealed a strong dependence on the molecular composition: below the *T*_g_, the activation energy decreased with TBS, indicating a transition toward a side-group reorientation-dominated mechanism, while above the *T*_g_, the TBS monomers participate in the process, despite PTBS lacking a β-relaxation. The coexistence of TBS and MMA monomers revealed a fundamental shift in relaxation dynamics manifested by the decoupling of α- and β- relaxations, which we analyzed via the double-percolation mechanism. Our findings offer new insights into polymer relaxation behavior and the relationship between the α- and β- relaxation mechanisms with implications in materials optimization.

## Graphical abstract

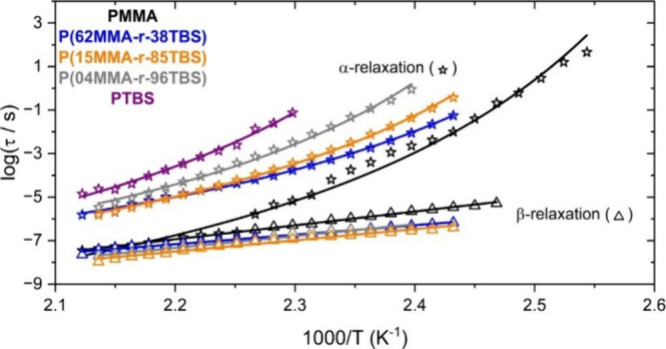

## 1 Introduction

In disordered (amorphous) polymers, the main event that determines a great range of properties, i.e., thermal, mechanical, electrical or dielectric, is the glass transition process. This process occurs over a short-range of temperatures signified by the glass transition temperature, *T*_g_, which most often is detected experimentally by a rapid change in the heat capacity of the sample upon cooling from the liquid state to the glassy state.^1^ It should be noted that the glass transition process is not a first-order phase transition, like the melting of a crystal that usually is accompanied by a step-change decrease in the specific volume or other thermodynamic state variables, but instead is a kinetic phenomenon.^2^ Therefore, the location of the *T*_g_ is time-dependent; thus, it depends upon the heating or cooling rates or the frequency of an externally applied dynamic disturbance.^3^ In the latter case, the process associated with the *T*_g_ is commonly called segmental or α- relaxation, since it rises due to the relaxation of short backbone segments (2–3 nm long) that interact with their neighboring segments resulting in cooperative motion.^4,5^ Depending upon the measurement temperature, the relaxation time of the α-relaxation ranges from fractions of a second to months and increases as the temperature decreases toward *T*_g_. Moreover, the α-relaxation’s temperature dependence strongly deviates from Arrhenius behavior that is characterized by a single activation energy, indicating that in this case the activation energy is temperature dependent and increases while approaching the *T*_g_ as the correlation length of the α-relaxation increases.^6–9^ To quantify the deviation from the Arrhenius behavior exhibited by the α-relaxation, Angell introduced the concept of fragility and associated it with the temperature dependence of viscosity or relaxation time.^10,11^ Structural aspects of disordered polymers, like backbone flexibility or the bulkiness of the side groups, and particularly their corresponding relative relationship, have been observed to impact fragility.^12^ The intricate relationship between molecular structure and segmental mobility of polymers remains a central yet evolving challenge in polymer science. Establishing a structure–property correlation becomes further perplexed in copolymers, where the resulting properties depend not only on the monomers but upon composition and spatial arrangement.

At temperatures lower than *T*_g_, i.e., in the glassy state, and depending on the polymer’s structure, more processes can be found and usually are referred to as secondary relaxations, and correspond to molecular motions on a shorter, more localized length scale. The most prominent secondary relaxation is the Johari–Goldstein or β_JG_-relaxation.^13^ The β_JG_-relaxation has been observed to relate with the α-relaxation, which implies that the β_JG_-relaxation is sensitive to the glass transition, and evolves with time by increasing the number of participating units, thus acting as a precursor of the α-relaxation below the *T*_g_.^14^ Moving from high to low temperatures, commonly the α-relaxation is merged with the β_JG_-relaxation and eventually decouples at ∼1.2*T*_g_ where the latter exhibits an Arrhenius temperature dependence and stays active at *T* < *T*_g_, i.e., in the glassy state. Several ideas have been discussed to explain the physical origin behind the β_JG_-relaxation in metallic glasses or disordered polymers, considering a degree of cooperative motions in string-like excitations occurring due to hopping of the participating molecules,^15–17^ and its relationship to physical aging.^18^ Goldstein originally suggested that the β_JG_-relaxation originates from ‘islands of mobility’ within the ‘frozen’ glassy state, indicating dynamic heterogeneity.^19^ Since then, many studies have examined variations of this idea, with recent works suggesting that the molecules participating in the β_JG_-relaxation are highly mobile and are part of a percolating cluster, forming a fragmented mosaic structure.^20^ Going from higher temperatures (above *T*_g_) to lower temperatures, this system has percolating immobile and mobile regions that correlate to the α- and β- relaxations, respectively, as proposed recently by Dyre.^21^ In the case of polymers, it is not possible to have independent atoms or molecules that can individually migrate even when the surrounding ‘matrix’ is frozen (sub-*T*_g_) as with metallic glasses or glass-forming small organic molecules. Instead, parts of the polymer chain, i.e., side groups or a small group of monomers that are shorter than the Kuhn length and do not constitute a segment (which refers to the α-relaxation) can be perceived as islands of mobility, where the segments are frozen, i.e., island of immobility in sub-*T*_g_ conditions. The relationship between the two processes and thus, the manifestation of the β-relaxation, relates to the system’s dimensionality and the spatial percolation of fast motions through the lowest energy barrier regions.^22,23^ Moreover, certain polymers display a well-defined β-relaxation (so-called *type-B*), whereas others do not (so-called *type-A*).^24^ Studies of binary mixtures of these two types have demonstrated that the β-process disappears when *type-B* molecules are diluted, and that these molecules contribute less to the β-process beyond a specific concentration threshold.^25^

In the present work, we systematically investigate the dielectric relaxation dynamics of random copolymers based on poly­(methyl methacrylate) (PMMA) and poly­(4-*tert*-butylstyrene) (PTBS) across various monomer compositions. The two polymers were chosen based on the variation between their *T*_g_ *s* by ∼20 °C. Moreover, PMMA exhibits two secondary relaxations, β- and γ-, while PTBS has none, showcasing an interesting combination to examine the Johari–Goldstein relaxation and investigate the structure–property relationship that governs random copolymers. By means of Differential Scanning Calorimetry (DSC) we found a strong dependence of the *T*_g_ on polymer structure, which deviates from the Fox equation. To elucidate the underlying mechanisms, we examined the α-relaxation and demonstrated the interplay between monomer bulkiness and composition using the framework of free volume to explain the observed deviation from the Fox equation. Additionally, we explored β-relaxation dynamics, identifying significant reductions in dielectric strength and activation energy as MMA content decreased. The strong dependence of the low-temperature component of β-relaxation on MMA concentration confirmed its origin within these monomers, while the high-temperature component of β-processes suggested the participation of TBS units, despite PTBS homopolymer lacking intrinsic β-relaxation. Finally, we observed a decoupling between the α- and β- relaxations in the presence of TBS which we investigated through the double-percolation mechanism. Reformulating Arrhenius and VFT equations, we derive a characteristic relaxation time scale at which the α- and β- relaxations are at their closest proximity without merging. We found that this characteristic relaxation time scale only depends on the fragility parameter and pre-exponential factors and surprisingly not on the activation energy of the β-relaxation or the Vogel temperature of the α-relaxation.

## 2 Methods

### 2.1 Chemical Synthesis

Azobis­(isobutyronitrile) (AIBN, 10 mg, 0.060 mmol, 1 equiv) was added to a 50 mL round-bottom flask sealed with a septum. The flask was vacuum purged with dry nitrogen 3 times. Then, monomers (MMA and TBS, 6 mmol, 100 equiv) and toluene (10 mL) were deoxygenated for 15 min by bubbling with dry nitrogen followed by addition to the flask. The reaction mixture solution was stirred at 100 °C for an hour. The crude product was diluted with additional toluene and precipitated in cold methanol to get a white product. Then, the product was dissolved in THF and reprecipitated into methanol. The final product was dried under vacuum at 40 °C to obtain white powder.

### 2.2 Chemical Characterization

The copolymer composition was determined by ^1^H nuclear magnetic resonance (NMR) spectroscopy. Polymers were dissolved at 5 mg/mL in deuterated chloroform (99.8%, Cambridge Isotope Laboratories, Inc.), and spectra were collected on a Bruker Avance III 500 MHz spectrometer. All NMR data analysis was performed with MestReNova software (Mestrelab Research). The weight-average molecular weight (*M*_*w*_), number-average molecular weight (*M*_*n*_), and dispersity (Đ) were characterized by gel permeation chromatography (GPC) using two 30 cm Agilent PLgel 5 μm Mixed-C columns operating at 35 °C, a Wyatt Optilab T- rEX differential refractive index (DRI) detector (25 °C, 658 nm wavelength), and a miniDAWN TREOS three-angle light scattering instrument (ambient temperature, 658 nm). Tetrahydrofuran (inhibitor-free HPLC, Sigma-Aldrich) was used as the mobile phase. *M*_*w*_ was measured by using light scattering. The specific refractive index increment *dn/dc* for each polymer was calculated using a weight-fraction average for PMMA (0.089 mL/g) and PTBS (0.185 mL/g) using the polymer composition determined from ^1^H NMR. The DRI elution time was calibrated with narrow-distribution polystyrene standards, from which the chain-length dispersity Đ was determined. Finally, *M*_*n*_ was calculated as *M*_*n*_ = *M*_*w*_ */Đ*. The samples were named based on their corresponding MMA-TBS mole percentages in the polymer, i.e., P­(*X*MMA-r-*Y*TBS) where *X* and *Y* are the MMA and TBS mole percentages, respectively. More information can be found in Table 1.

**Table 1:** Chemical Information of the Synthesized Polymers

sample name	feed mole ratio of MMA (%)	*M*_*n*_ (kDa)	Đ	*F*_MMA_ (%)	MMA (% w/w)
PMMA	100	9.5	1.50	100	100
P(81MMA-r-19TBS)	90	14.7	1.65	81.3	73.1
P(62MMA-r-38TBS)	80	12.2	1.66	62.6	51.1
P(47MMA-r-53TBS)	70	11.6	1.80	46.9	35.6
P(35MMA-r-65TBS)	60	16.4	1.78	34.6	24.8
P(26MMA-r-74TBS)	50	11.9	1.80	25.5	17.6
P(15MMA-r-85TBS)	40	11.6	1.88	15.1	10.0
P(13MMA-r-87TBS)	30	13.5	1.89	13.3	8.7
P(04MMA-r-96TBS)	10	14.2	1.79	3.6	2.3
PTBS	0	14.3	1.98	0	0

### 2.3 Sample Preparation

#### Compression Molding

Uniformly cylindrical specimens with a diameter of 2.54 cm were prepared via compression molding (Teach-line Platen Press 200E hot press) using a custom-made stainless-steel mold. Kapton tape protectors were applied on both the top and bottom faces of the mold and then filled with the synthesized polymer in powder form. Initially the mold was placed in the hot press under no pressure for 15 min at approximately 30 °C above the *T*_g_ as determined by DSC in powder samples. Twenty MPa of mechanical stress were applied for 15 min after which, the heating was turned off without relieving the pressure. Finally, the mold was removed from the hot press when the temperature dropped below 80 °C, yielding specimens with thicknesses in the order of 0.4 to 1.3 mm.

### 2.4 Physical Characterization

#### Differential Scanning Calorimetry

DSC was employed to measure the *T*_g_ of the developed polymer samples after compression molding. The measurements were performed using a Discovery DSC 2500 provided by TA Instruments, equipped with an RCS90 cooler. The DSC furnace chamber was constantly flushed with nitrogen. Typically, between 2 and 8 mg of material after compression molding were placed into aluminum hermetically sealed pans. The DSC protocol included a sequence of heating – cooling – heating at 10 °C/min, over the temperature range of −30 to 200 °C. All reported *T*_g_ values correspond to the midpoint *T*_g_ and were calculated on heating using available tools in the TRIOS software. The obtained *T*_g_ values were plotted against the weight concentration values of the examined random PMMA/PTBS copolymers and compared with the Fox equation:

$$T_{g}=\frac{T_{g1}T_{g2}}{w_{1}T_{g2}+w_{2}T_{g1}}$$ 1

where *T*_*g1*_ and *T*_*g2*_ correspond to the *T*_g_ values of the homopolymers PMMA and PTBS obtained from DSC, respectively. The *w*_1_ and *w*_2_ values correspond to the weight fractions of MMA and TBS monomers respectively, as obtained from the chemical synthesis. Since the *T*_g_ values of the random copolymers deviate strongly from the Fox equation, the Gordon–Taylor equation was also employed, with K being a fitting parameter, as seen below:

$$T_{g}=T_{g1}+\left(T_{g2}-T_{g1}\right)\left(\frac{Kw_{2}}{w_{1}+Kw_{2}}\right)$$ 2

#### Broadband Dielectric Spectroscopy

BDS was employed to unravel the relaxation map of the polymer samples under study. The apparatus consists of an Alpha-A high performance modular measurement system and a Phecos temperature-controlled chamber that has an integrated dielectric cell, all provided by Novocontrol Technologies, Germany. The voltage amplitude V_rms_ of the applied field was kept constant at 1.0 V, and the frequency range examined was 10^–1^ to 10^6^ Hz. The measurements were conducted in a parallel-plate capacitor configuration, with two external gold-plated electrodes and the sample in between them. The isothermal scans were measured in the temperature range of −48 to 195 °C, in steps of 3 °C (with ± 0.1 °C accuracy). Most often the dielectric data are analyzed via the complex dielectric permittivity function ε* which is defined as follows:

$$\varepsilon^{*}\left(\omega \right)=\varepsilon^{\prime }\left(\omega \right)-i\varepsilon^{\prime \prime }\left(\omega \right)$$ 3

where ε′ and ε″ are the real and imaginary parts of dielectric permittivity and ω is the angular frequency equal to *2πf* with *f* being the frequency of the externally applied electric field. The isothermal dielectric spectra can be analyzed using the Havriliak–Negami (HN) function in its complex permittivity form for a sum (superposition) of *N* dipolar processes and conductivity contribution:

$$\varepsilon_{HN}^{*}\left(\omega \right)=\varepsilon_{\infty }+\overset{N}{\sum }\frac{\varepsilon_{s}-\varepsilon_{\infty }}{{\left[1+{\left(i\omega \tau_{HN}\right)}^{k}\right]}^{n}}+\frac{\sigma_{0}}{i\varepsilon_{0}\omega^{s}}$$ 4

with ε_*s*_ and ε_∞_ being the static and infinite frequency permittivity values, respectively according to $\varepsilon_{s}=\underset{\omega \to 0}{\lim}\;\varepsilon^{\prime }\left(\omega \right)$ and $\varepsilon_{\infty }=\underset{\omega \to \infty }{\lim}\;\varepsilon^{\prime }\left(\omega \right)$ . σ_0_ corresponds to the electrical conductivity, ε_0_ is the dielectric constant of vacuum (8.854 × 10^–12^ F/m) and exponent *s* ≤ 1. As τ_*HN*_, the value of the isothermal HN relaxation time is represented. Shape parameters *k* and *n* correspond to the symmetrical and asymmetrical distribution of relaxation times respectively and vary between 0 < k, k_*_ *n < 1*; for *k = n =* 1, eq 4 reduces to the Debye model for a single relaxation time.^26^ The Debye model is a fundamentally important model that describes the ideal case of a dipolar process characterized by a single relaxation time. However, experiments have shown that dipolar processes are most often broader, symmetrical or asymmetrical, resulting in a distribution of relaxation times rather than a singular value.^26–28^ When *n ≠ 1* the characteristic relaxation time of the dielectric process is not equal to the relaxation time τ, which is equal to the reciprocal value of the angular frequency where the loss peak is observed, i.e., τ_*HN*_ ≠ τ, as shown by eq 5:^29^

$$\tau =\tau_{HN}{\left[\frac{\sin\left(\frac{kn\pi }{2+2n}\right)}{\sin\left(\frac{k\pi }{2+2n}\right)}\right]}^{1/k}$$ 5

To examine the temperature dependence of the α-relaxation, the relaxation times calculated from HN fittings are fitted with the Vogel–Fulcher–Tammann (VFT) equation as shown below in eq 6:

$$\tau =\tau_{0,\alpha }\exp\left[\frac{DT_{V}}{T-T_{V}}\right]$$ 6

where τ_0, α_ corresponds to the value of the relaxation time at infinite temperature. *T* is the absolute temperature and *T*_*V*_ is the Vogel temperature or ideal *T*_g_ and is expected to be 30 to 70 K below the actual calorimetric *T*_g_.^30,31^ Here, *T*_*V*_ was assumed to be *T*_g_ – 50 K. *D* is a dimensionless parameter that is associated with the steepness (fragility) of the segmental relaxation and can be related to the depths and density of minima in the potential-energy landscape of the glassy polymer.^10,32^ The VFT fitting parameters *D* and *T*_*V*_ as well as the *T*_g_ value obtained from DSC measurements are used to calculate the fragility parameter *m*, as seen below:^33^

$$m=\frac{DT_{V}T_{g}}{{\left(T_{g}-T_{V}\right)}^{2}\ln\left(10\right)}$$ 7

The dielectric strength relates to the number density of dipoles (*N/V*) through the generalized Debye theory of Kirkwood and Fröhlich, presented in eq 8, that contributes toward a single dipolar relaxation.^34,35^

$$\Delta \varepsilon \approx \frac{1}{3\varepsilon_{0}}Fg\frac{{\left\langle \mu \right\rangle }^{2}}{k_{B}T}\left(\frac{N}{V}\right)$$ 8

where ⟨μ⟩ is the mean dipole moment related to the specific dipolar process and *g* is the Kirkwood – Fröhlich correlation factor that describes the interaction between dipoles with respect to the ideal case of noninteracting dipoles (*g* = 1). *F* is the Onsager factor that describes the internal field effects and *k*_*B*_ is the Boltzmann constant (8.617 × 10^–5^ eV/K) and *T* is the absolute temperature.

Finally, like the VFT equation presented earlier for the α-relaxation, the secondary relaxations (γ-relaxation and β-relaxation) most often follow the Arrhenius law which is shown in eq 9:

$$\tau =\tau_{0,x}\exp\left[\frac{E_{A}}{k_{B}T}\right]$$ 9

where τ_0, *x*_ corresponds to the value of the relaxation time at infinite temperature, and *x* is either β or γ to distinguish between β- or γ- relaxations, respectively. *E*_*A*_ is the activation energy of the dielectric process and *T* is the absolute temperature.

## 3 Results and Discussion

### 3.1 Chemical Synthesis

(Methyl methacrylate), MMA, and (4-*tert*-butylstyrene), TBS, were used to prepare P­(MMA-r-PTBS) random copolymers in various monomer ratios, as shown in Figure 1a. The random copolymers were synthesized by thermally initiated radical polymerization in the presence of radical initiator, AIBN. The ^1^H NMR spectra of random copolymers are shown in Figure 1b, where the aromatic protons from TBS are displayed at 7.26–6.1 ppm and methoxy protons of MMA are located at 3.48 ppm. According to ^1^H NMR, the signal from TBS increases and the signal from MMA decreases as the feed amount of TBS increases. Using the integration ratio of these two peaks, we calculated the mole ratio of MMA in the copolymer. The smallest MMA weight percentage in the copolymer was 2.3% w/w when the feed contained 10 mol % MMA, and the largest MMA content in the copolymer was 73.1% w/w, from a feed containing 90 mol % MMA. The content of MMA in each copolymer is smaller than in the feed because of differences in monomer reactivity. The reactivity ratio was calculated by curve-fitting to the equation ( $F_{1}=\frac{r_{1}f_{1}^{2}+f_{1}f_{2}}{r_{1}f_{1}^{2}+2f_{1}f_{2}+r_{2}f_{2}^{2}}$ ), where *F* is the mole fraction of component *i* in the polymer, *f* is the mole fraction of component *i* in the monomer feed, *r* is reactivity ratio; this equation was numerically integrated up to the gravimetrically determined conversion (∼40%) for each polymerization, yielding reactivity ratio estimates of 4.3 for TBS and 0.34 for MMA. The molecular weights and distributions of each polymer were determined by GPC presented in Figure 1c. The MMA-TBS copolymers showed similar molecular weights (*M*_*n*_ = 13 ± 4 kg/mol) with similar chain length dispersities (1.5–2.0).

**Figure 1:**
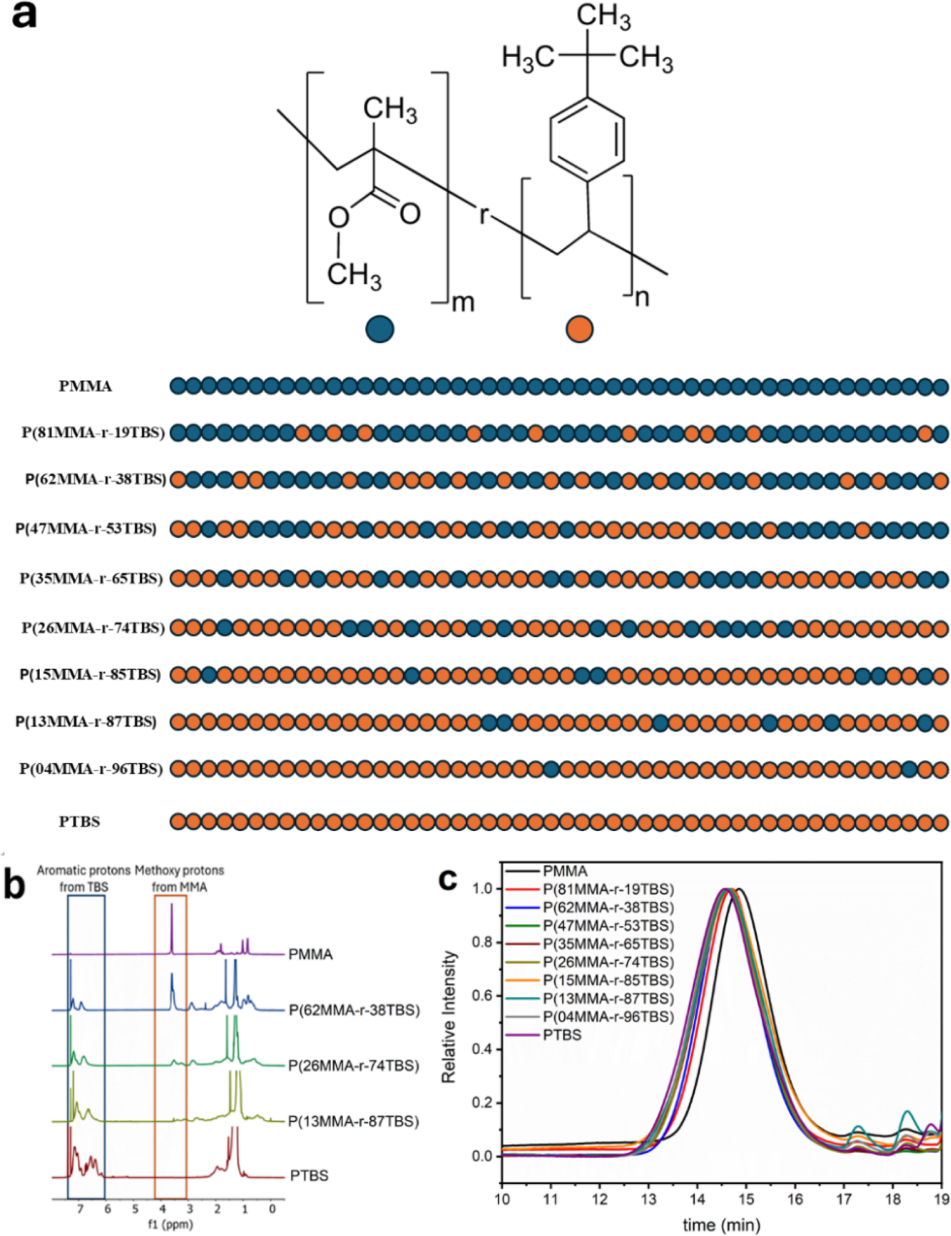
Chemical structure and synthesis. (a) Chemical structure of developed copolymers including a schematic representation of the random sequence of the two monomer units based on the mole concentration of each monomer; inspired by Taylor et al.^36^ The reaction conversion was ∼40% to prevent high product viscosity, thus the composition varies with the reactivity ratio. The polymer sequence is not expected to be gradient but random.^37^ (b) ^1^H NMR spectra and (c) GPC traces of the random copolymers and the two homopolymers.

### 3.2 Calorimetric *T*_g_ and Segmental Dynamics

The first goal of our study was to examine the *T*_g_ of the developed copolymers and unravel how it relates to the polymers’ chemical structure. Toward that purpose, we employed DSC and indeed observed a strong structure-dependence of *T*_g_, with values increasing from 122.5 to 142.5 °C with the progressive addition of the ‘bulkier’ TBS. Our DSC measurements show a single *T*_g_ for all the samples, indicating no evidence of phase separation.^38^ The *T*_g_ values strongly deviate from the typical Fox behavior presented in eq 1 and are best described by the Gordon–Taylor function described by eq 2 with a fitting parameter *K* value equal to ∼0.053. In random copolymers, the Gordon–Taylor parameter *K* is a semiempirical fitting parameter that determines the relative mobility and the intermolecular interaction between the two monomers that can be associated with hydrogen bonding, free volume or rigidity differences. In theory, *K* is linked to the densities and expansion coefficients or the heat capacities of the individual homopolymers, although deviations from this relationship are frequently observed.^39,40^ When the *K* values are equal to the ratio between the *T*_g_ *s* of the two homopolymers (in this case, ∼0.952), the Fox equation is recovered.^41^ In our system, it is evident in Figure 2a that the *T*_g_ of the random copolymers, as determined by DSC, is essentially the same with the PMMA homopolymer (∼122.5 °C) in the composition range of 25% to 100% MMA (gray shaded area), a striking observation that essentially implies that the TBS monomers appear not to participate in the calorimetric glass transition. However, we will subsequently demonstrate that this is not the case and that the underlying mechanisms involved are more complex than this simplified perspective suggests. At lower MMA concentrations, the *T*_g_ values suddenly increase significantly, until the PTBS homopolymer *T*_g_ value is achieved.

**Figure 2:**
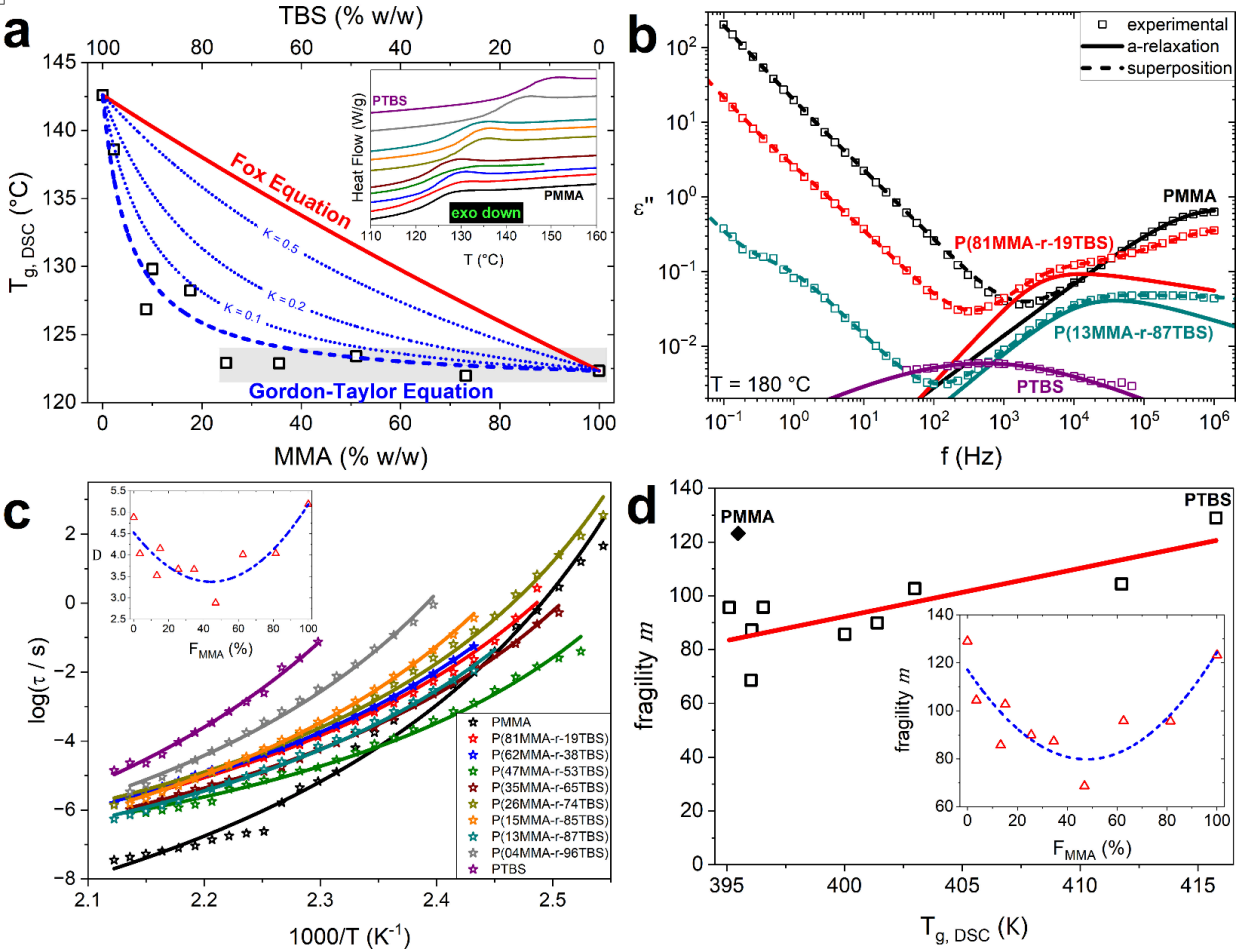
Calorimetric *T*_g_ and segmental dynamics. (a) Calorimetric *T*_g_ (open symbols) obtained from the 2^nd^ thermal cycle of DSC measurements (shown inset) presented against the MMA weight concentration and fitted with the Fox (red line) and Gordon–Taylor (blue dashed line) equations, described in eqs 1 and 2, respectively. With blue dotted lines, Gordon–Taylor fits for various K values are presented for comparison. (b) Dielectric response of selected samples as represented via the imaginary part of dielectric permittivity shown in eq 3 as a function of frequency at 180 °C with HN fits given in eq 4 where the α-relaxation is separated for clarity (bold line). The superposition (dashed line), i.e., linear sum, of the various contributions as described by the HN function, are shown to describe adequately the experimental values (open symbols). In the case of the PMMA homopolymer, at this temperature the α- and β-relaxation processes are merged. (c) Relaxation time, τ, of the α-relaxation (open symbols) plotted versus reciprocal temperature and fitted with the VFT function (lines) as shown in eq 6 with the fitting parameter *D* given as an inset (red open triangles) fitted with a Gaussian function (dashed blue line). (d) Fragility parameter *m* described by eq 7 plotted against the calorimetric *T*_g_ and fitted with a line (apart from the PMMA sample denoted with a full symbol). As inset, the fragility parameter *m* is shown as a function of the MMA mole concentration fitted with a Gaussian function (dashed blue line).

One aspect to consider as a contributing factor on the calorimetric *T*_g_ of the random copolymers, is the relationship between the tacticity of PMMA and the addition of TBS monomers. The calorimetric *T*_g_ of PMMA homopolymers strongly depends upon monomer tacticity, with isotactic (i-PMMA), atactic (a-PMMA), and syndiotactic (s-PMMA) yielding *T*_g_s at approximately 50 °C, 105 °C, and 125 °C, respectively. When PMMA homopolymer is synthesized via free radical polymerization at 100 °C in toluene, it commonly yields PMMA with high syndiotacticity.^42,43^ Following the same synthetic route, our PMMA homopolymer is 53% syndiotactic, 38% atactic, and 9% isotactic according to NMR, with its calorimetric *T*_g_ being 122.3 °C as measured by the DSC. According to a recent study,^44^ the insertion of a monomer disrupts the conformational structure of the homopolymer, thus affecting its tacticity. In the present case, it is possible that the addition of TBS leads to the local destruction of the conformational syndiotacticity of s-PMMA, resulting in a-PMMA blocks. In such case, the coexistence of bulky TBS units (high *T*_g_) with a-PMMA blocks (low *T*_g_), would result into two competing effects that render the average calorimetric *T*_g_ of the random copolymer similar to s-PMMA (PMMA homopolymer).

To further elucidate how the *T*_g_ relates to composition and determine which are the underlying mechanisms at a molecular level, we examined the dynamics of its corresponding process (α-relaxation) by means of Broadband Dielectric Relaxation (BDS).^45,46^ In the present analysis, the dielectric response of the copolymers under study were investigated via the complex dielectric permittivity function, as seen in eq 3. Figure 2b shows the imaginary part of dielectric permittivity as a function of frequency at 180 °C for selected samples. It is evident that the PMMA homopolymer exhibits higher dielectric losses among the presented samples, which is attributed to its greater polarizability, particularly when compared to the PTBS homopolymer.^47^ The dielectric spectrum of the PTBS homopolymer above the *T*_g_ is included in the Supporting Information in Figure S1. Therefore, by incorporating TBS into the random copolymers, the dielectric losses decrease until the lowest values are reached, corresponding to the PTBS homopolymer. According to Figure 2b, the main dipolar feature of the examined samples at 180 °C is the α-relaxation process, which is highlighted for clarity with solid lines that are described via the Havriliak–Negami (HN) function presented in eq 4. The isothermal value of the relaxation time, τ, that corresponds to the α-relaxation for each sample is equal to τ = 1/ω_*max*_ and relates to the Havriliak–Negami characteristic relaxation time τ_*HN*_ via eq 5.

It is evident in Figure 2c that the α-relaxation dynamics change significantly as a function of copolymer composition. Although separated by about 20 °C given the difference of their corresponding *T*_g_ *s*, both PMMA and PTBS exhibit a strong dependence on temperature in their relaxation times, that can be quantified through the VFT equation, shown in eq 6. The fitting parameter *D*, from the VFT equation is presented in the inset of Figure 2c. The steepness of the α-relaxation is associated with the backbone flexibility and the bulkiness of the side groups, better described by fragility calculated via eq 7. Both PMMA and PTBS homopolymers exhibit high fragility values (*m* ≈ 125) ascribed to their side groups.^12^ Although both side groups are bulky, when combined randomly to form copolymers, we observe a strong reduction in the fragility values that are dependent upon chemical structure, as shown in Figure 2d inset. In PMMA homopolymers, fragility has been shown to increase with randomness in tacticity.^48,49^ Here, for copolymers, we observed that fragility values decreased with increasing TBS content until a near 1:1 mol ratio. In other words, fragility decreased with increasing randomness going from PMMA homopolymer to 50 MMA mole %. The observed difference between tacticity effects on fragility in homopolymers and random copolymers, indicates that in the latter, the underlying mechanisms extend further than tacticity.

To rationalize our results, we need to understand two aspects; first, that the TBS monomer is bulkier than MMA, and second that the extra methyl units at the end of the phenyl group act as molecular spacers (i.e., plasticizers) that overall decrease fragility. Therefore, if we start with the PMMA homopolymer and progressively add TBS monomers randomly, the bulky phenyl groups with the extra methyl units would be expected to frustrate local packing and increase free volume. On the other hand, if we start from the PTBS homopolymer and gradually add MMA units randomly, we expect increased polymer flexibility due to the smaller, more flexible MMA units. Both cases are presented schematically in Figure 3a,b, respectively. Figure 3 is intended as a simplified illustration to convey our key points; therefore, it is not meant to represent the microscopic reality with complete accuracy. These two competing effects explain the significant reduction in fragility that ‘peaks’ when the two monomers are close to 1:1 in mole ratio (50%), presented in Figure 2d inset. The two competing effects can also be used to explain the *T*_g_ behavior observed in Figure 2a. While the addition of TBS into the PMMA chain increased ‘bulkiness’ those effects were counteracted by changes in local packing, and hence free volume. In contrast, at higher TBS content (75% to 100% w/w), chain packing is less frustrated, free volume decreases, and works additively with the high content of bulky TBS monomers to increase *T*_g_ in an accelerated manner, resulting in an observed so-called ‘super-Fox’ behavior, i.e., where *T*_g_ increases faster with composition than what the Fox equation describes.

**Figure 3:**
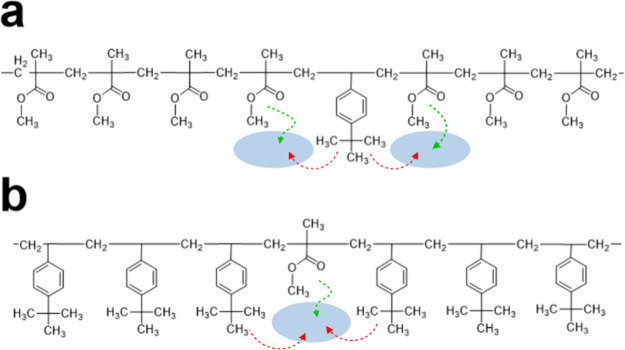
Schematic representation of the free volume mechanism in the random copolymers. (a) Presence of TBS in a PMMA chain. (b) Presence of MMA in a PTBS chain. The shaded blue areas correspond to hypothesized free volume regions generated by the variation in bulkiness between the two monomers, and the arrows are associated with additional movements of the monomers close to these regions (green for MMA and red for TBS). Created in BioRender. Drakopoulos, S. (2025) https://BioRender.com/inq9jz9.

Moreover, we observed a somewhat linear relationship between fragility and *T*_g_ presented in Figure 2d, where the PMMA homopolymer was observed to deviate from this trend. To understand this result, we first need to remember that *T*_g_ depends strongly on monomer bulkiness and chain flexibility,^50,51^ while the relative relationship between the two correlates with fragility.^12^ Therefore, in the absence of TBS units, i.e., PMMA homopolymer, the less bulky MMA monomers result in a decreased *T*_g_ and efficient molecular packing, i.e., decreased free volume, resulting to increased fragility values. The remaining samples showcase the observed linear relationship indicating how restricted free volume in the presence of bulky monomers, enhances *T*_g_. At low fragility values, i.e., high free volume, the backbone is more flexible, which allows for lower *T*_g_ *s* despite the presence of bulky TBS monomers in the polymer chain. On the other hand, as fragility values increase, i.e., restricted free volume, the presence of the bulky TBS monomers appears to play a more prominent role and thus leads to an increase of *T*_g_.

### 3.3 Secondary Relaxations

In addition to the α-relaxation, more dipolar processes were identified and attributed to the β- and γ- relaxations, respectively, shown in the complete relaxation map of all the samples under study in Figure S2 in Supporting Information. As discussed earlier in detail, the α-relaxation of the random copolymers originates from segmental motions of the backbone that consists of both the MMA and the TBS monomers. However, the secondary relaxations are expected to originate from MMA alone, since PTBS does not exhibit any relaxation process other than the α-relaxation.^52^ At temperatures in the range of −48 to 72 °C we observe the γ-relaxation (Figure S2) that is attributed to isolated rotation of the main-chain methyl group,^53^ and its relaxation time appears to vary with the MMA concentration. At greater time scales, PMMA exhibits a strong and asymmetric β-relaxation that is known to exhibit a double Arrhenius behavior with a crossover close to the *T*_g_, above which the activation energy increases significantly.^54,55^ The origin behind the β-relaxation of PMMA has been attributed to the reorientation of side groups and more specifically 180° flips of the planar ester side-group as studied by NMR.^56,57^ In addition, the contribution of small parts of the backbone has also been reported,^58^ that constitute the β-relaxation a Johari–Goldstein process.^54,59^

Figure 4, which shows the β-relaxation observed in our systems, is the basis for our discussion, with a specific focus on the low temperature component of the process, i.e., below *T*_g_. Figure 4a presents the imaginary part of dielectric permittivity as a function of temperature at 100 Hz for all samples under study, where both the α- and β-relaxations are shown as two, well-defined peaks. It is evident that the intensity of the β-relaxation reduced as the MMA content decreased. It can be better quantified by expressing the dielectric strength, Δε, of the β-relaxation against the MMA mole concentration in the system presented in Figure 4b, where a strong linear relationship was found. The linear relationship between the β-relaxation and the MMA mole concentration strongly indicates that the process arises only from the MMA monomers, since the number density of dipoles (*N/V*) is proportional to the MMA mole concentration, as expressed through the generalized Debye theory of Kirkwood and Fröhlich in eq 8. Therefore, the ester group that gives rise to the β-relaxation in the PMMA homopolymer, acts as a dielectric probe monitoring the local dynamic environments in the random copolymers. In PMMA homopolymers, the β-relaxation exhibits a weaker dependence on tacticity than the α-relaxation. One study has shown that, for PMMA homopolymers, increasing randomness in tacticity leads to a higher activation energy and a longer relaxation time for the β-relaxation.^60^ Here, for random copolymers, we observed the opposite effect, as with fragility values of the α-relaxation presented earlier. In our systems, the temperature loss peak position of the β-relaxation shifts to lower temperatures with decreasing MMA content, as seen in Figure 4a. This can be corroborated by Figure 4c where a drastic reduction in the relaxation times of the process is observed when less MMA is present, implying that the process is facilitated when the MMA units are disrupted by TBS monomers. The facilitation of the β-relaxation can be further supported by Figure 4d which shows that the activation energy, *E*_*A*_, significantly reduces with TBS content (by almost 60%). Essentially, from the decreasing *E*_*A*_ values and relaxation times, we suggest that the TBS monomers are disrupting the MMA blocks, gradually suppressing the local movements of the backbone, and reducing the Johari–Goldstein “character” of the process.^61^ Therefore, at low MMA content, the β-relaxation likely originates only from the reorientation of the methyl methacrylate group.^58^

**Figure 4:**
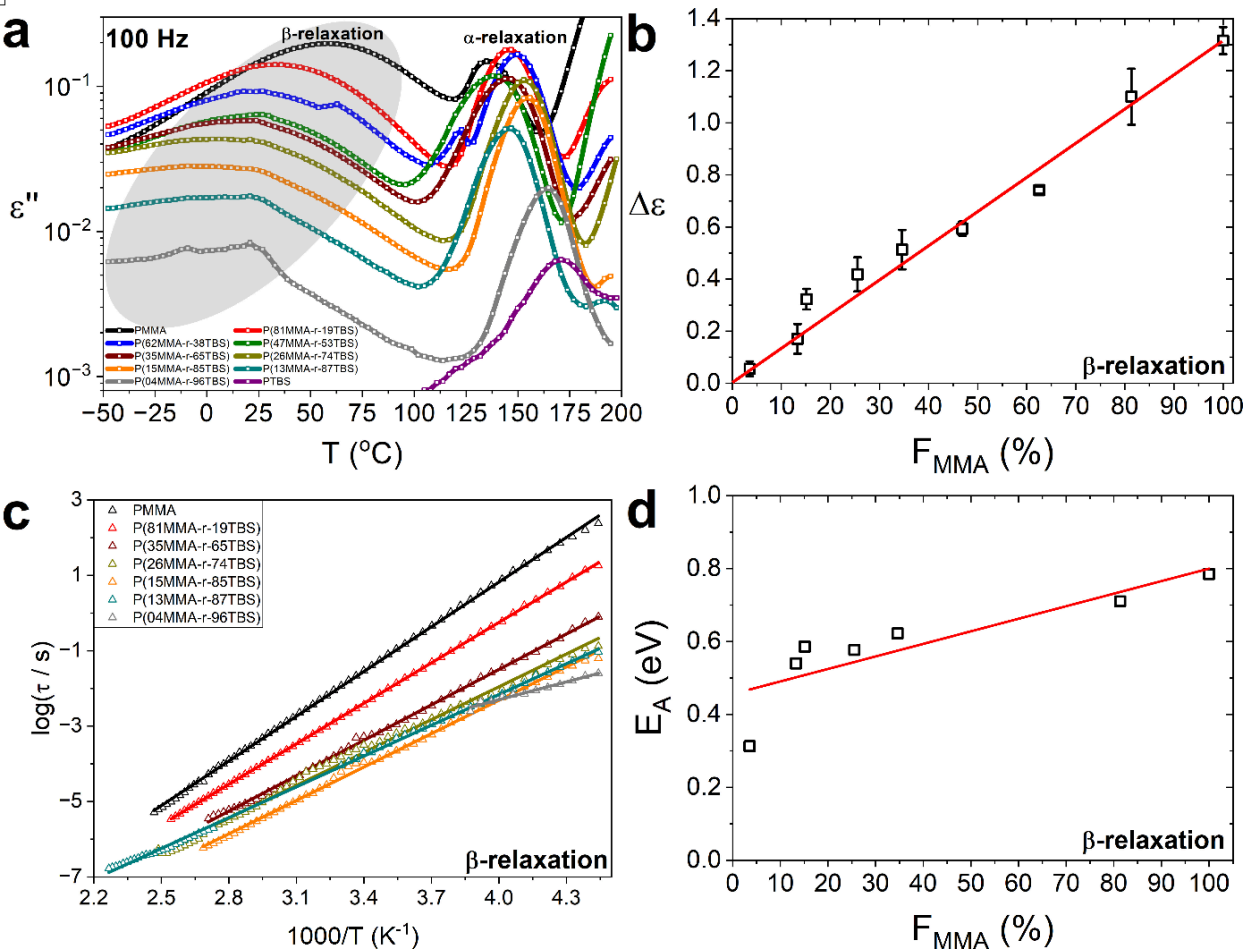
Low-temperature component of the β-relaxation and its relationship to locality/cooperativity. (a) Imaginary part of dielectric permittivity as a function of temperature at 100 Hz for all samples under study, with the β-relaxation highlighted. The minor experimental errors we observe occur in only a few cases below the calorimetric *T*_g_, and we attribute them partially to the nonpolar nature of TBS. (b) The Δε values (open symbols) obtained from the HN fittings are presented as a function of the MMA mole concentration and fitted with a line with zero intercept to represent that the PTBS homopolymer does not contribute toward the β-relaxation. The Δε can be described by the generalized Debye theory of Kirkwood and Fröhlich, presented in eq 8. The Δε values are averages over several temperatures and the error bars correspond to the standard deviation. Averages across different temperatures were chosen to highlight that this observation is applicable throughout a temperature range and not at just one temperature. (c) Relaxation time, τ, of the low-temperature β-relaxation (open symbols) against the reciprocal temperature and fitted with the Arrhenius law (lines) as shown in eq 9. (d) The *E*_*A*_ values (open symbols) are plotted against the MMA mole concentration and fitted with a line.

### 3.4 Dissociation between α- and β- Relaxations in the Presence of TBS

Moving to higher temperatures (above the *T*_g_), the relaxation times of the β-relaxation are described by a much shorter variation (∼1 order of magnitude) between samples, compared to low temperatures, as shown in Figure S2, while they do not exhibit a trend in the activation energy. The drastic increase in the *E*_*A*_ values upon crossing the calorimetric *T*_g_ suggests that the free volume allows for greater mobility from parts of the backbone to contribute to the process. Figure 5a shows that samples consisting of 100%, 62%, and 4% MMA are described by similar β-relaxation dynamics (*E*_*A*_ and τ values), while the PMMA homopolymer exhibits the typical merger of the two processes. We established in Figure 2 that both monomers contribute to segmental motions and we know that the β_JG_-relaxation acts as a precursor of the α-relaxation below the *T*_g_.^14^ Therefore, it should not come as a surprise that TBS monomers might participate in the high temperature component of the β-relaxation case of the random copolymers, even if the PTBS homopolymer does not exhibit a β-relaxation. Therefore, all the above suggest that the high temperature component of the β-relaxation might be a Johari–Goldstein process, even in the case of the random copolymers. Further experimental investigations and analysis may be necessary before definitive conclusions can be drawn on this matter.

**Figure 5:**
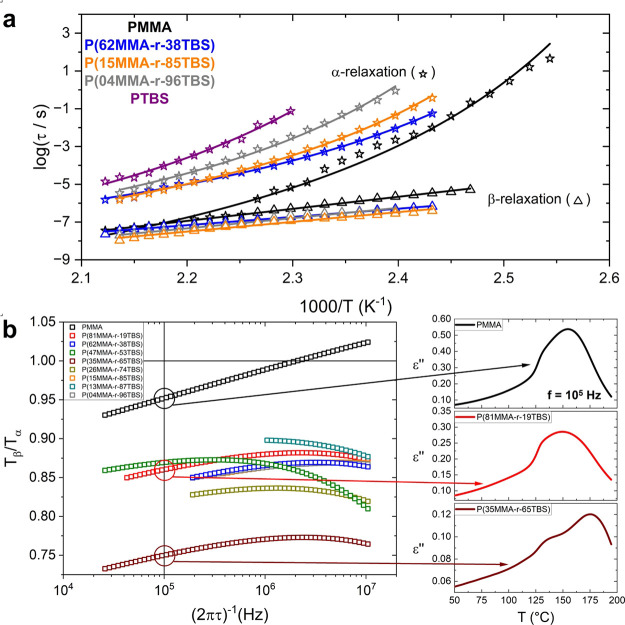
High-temperature unmerging of the β- and α- relaxation processes in the presence of TBS. (a) Part of the relaxation map for selected samples depicting the α-relaxation and the high-temperature component of the β-relaxation that highlights the unmerging between the two processes upon the addition of the TBS monomer. (b) (Left) Ratio between the β- and α- relaxation temperature loss peak positions against reciprocal relaxation time, calculated based on the VFT and Arrhenius law fittings. The merging point between the two relaxation processes (*T*_β_ = *T*_α_) and the reciprocal relaxation time equal to 10^5^ Hz are highlighted with straight lines. (Right) The manifestation of the β-relaxation at *f* = 10^5^ Hz is shown for three samples that exhibit three different behaviors. At this frequency, the β-relaxation compared to the α-relaxation peak is shown as a wing for PMMA, a shoulder for P­(81MMA-r-19TBS), and a well-defined peak for P­(35MMA-r-65TBS).

As presented in the complete relaxation map in Figure S2 and shown for specific cases in Figure 5a, only the PMMA homopolymer exhibits the expected merging of the α- and β- relaxations,^62^ while the copolymers do not—this is a striking difference in dynamic behavior. The crossover temperature between the two relaxations in a homogeneous material depends upon its thermal treatment in the glassy state,^63^ which in our case is irrelevant since all the samples were treated the same way. Moreover, increased randomness in the tacticity of PMMA may lead to a dissociation between the α- and β- relaxations.^60^ However, as discussed earlier, our random copolymer system differs from PMMA homopolymers with varying tacticity in respect to fragility and β-relaxation dynamics. Thus, while increased randomness in the tacticity of PMMA cannot be excluded as a contributing factor, it alone is not expected to fully account for the observed dissociation between the α- and β-relaxations in our PMMA–PTBS random copolymers. The addition of TBS strongly affects the dynamics of the two relaxations, as it significantly slows down the α-relaxation, and leads to a dissociation between the two processes. Therefore, our findings are indicative of a complicated role of TBS in the relaxation dynamics.

To shed more light toward the observed dissociation of the β- and α- relaxations with the addition of TBS, we considered recent work that studied the relationship between the percolating immobile and mobile regions by examining the ratio between their corresponding temperature loss peak positions, i.e., *T*_*Pm*_ for mobile and *T*_*Pim*_ for immobile regions, respectively. In this study,^23^ the authors observed that when this ratio (*T*_*Pm*_ */T*_*Pim*_) is below roughly 0.85, the mobile- and immobile-particle percolation transitions are well separated and thus the β-relaxation is well-defined and manifested as a ‘shoulder’ or a ‘peak’, whereas the β-relaxation is absent or at most a ‘wing’ for percolation-temperature ratios larger than 0.85. According to the double-percolation picture, there are three percolating regions: 1) at low temperatures (or high frequencies) where only the mobile particles can percolate, 2) at high temperatures (or low frequencies) where only the immobile particles can percolate and, 3) at temperatures (or frequencies) in between where a double percolation scenario occurs such that both the immobile and the mobile particles percolate simultaneously. Therefore, the greater the disparity between *T*_*Pm*_ and *T*_*Pim*_, the greater the temperature (or frequency) range at which double percolation occurs. On the other hand, when the two overlap, i.e., *T*_*Pm*_ = *T*_*Pim*_, this translates to two separate regions where only one type of percolating particles is allowed (the double percolation is not permitted). Moreover, in physical terms, low values of the *T*_*Pm*_ */T*_*Pim*_ ratio showcase narrower peaks indicating small widths of the activation energy distribution of the molecular processes,^23^ which relates to narrower distributions of relaxation times.^64–66^

To examine this effect in our systems, and considering that the mobile- and immobile-particle percolation transitions correspond to the β- and the α- relaxations, respectively,^21^ we instead examined the *T*_β_ */T*_α_ ratio across many frequencies, as calculated from the Arrhenius and VFT fittings of the two processes, and presented in Figure 5b. The PMMA homopolymer exhibits significantly higher *T*_β_ */T*_α_ ratio values than the copolymers and as expected reaches a point where *T*_β_ */T*_α_ = 1 which corresponds to the crossover temperature. Another significant variation in the presented *T*_β_ */T*_α_ ratio representation between the PMMA homopolymer and the random copolymers is that the curves appear differently; all samples but PMMA exhibit a fully formed or partially formed peak that corresponds to a certain τ value, i.e., τ_*p*_. To understand this relationship, we need to review Figure 5b and understand what is being presented. The ratio *T*_β_ */T*_α_ essentially encloses information from both the Arrhenius and VFT equations, presented in eq 6 and eq 9, respectively. Therefore, the two equations can be reordered and *T*_β_ */T*_α_ can be rewritten as presented in eq 10:

$$\frac{T_{\beta }}{T_{\alpha }}=\left(\frac{E_{A}/k_{B}}{\ln\left(\tau \right)-\ln\left(\tau_{0,\beta }\right)}\right){\left(T_{V}+\frac{DT_{V}}{\ln\left(\tau \right)-\ln\left(\tau_{0,\alpha }\right)}\right)}^{-1}$$ 10

where τ_0, β_ and τ_0, α_ correspond to the values of the relaxation time at infinite temperature, i.e., pre-exponential factors, for the β- and the α- relaxations, respectively, and τ being the variable. At this point, we note that the applicability of the VFT function has been proven to be insufficient over a great range of temperatures;^67,68^ however, for the examined temperature range presented here, the VFT function describes adequately the data and thus it is appropriate for further analysis. Moreover, both the VFT and the Arrhenius functions are the most widely used equations to describe the temperature dependence of the segmental and secondary relaxations, respectively. Since the PMMA homopolymer and the copolymers exhibit either an absence or presence of *T*_β_ */T*_α_ extrema (maxima or minima), we investigate further by calculating the first order derivative with *ln­(τ)* shown in eq 11:

$$\frac{d}{d\ln\left(\tau \right)}\left(\frac{T_{\beta }}{T_{\alpha }}\right)=\left(\frac{E_{A}}{k_{B}T_{V}\left(\ln\left(\tau \right)-\ln\left(\tau_{0,\beta }\right)\right)}\right)\left(\frac{\frac{D}{{\left(\ln\left(\tau \right)-\ln\left(\tau_{0,\alpha }\right)\right)}^{2}}-\frac{1+\frac{D}{\ln\left(\tau \right)-\ln\left(\tau_{0,\alpha }\right)}}{\ln\left(\tau \right)-\ln\left(\tau_{0,\beta }\right)}}{{\left(1+\frac{D}{\ln\left(\tau \right)-\ln\left(\tau_{0,\alpha }\right)}\right)}^{2}}\right)$$ 11

In the case of the random copolymers an extremum is observed, therefore $\frac{d}{d\ln\left(\tau \right)}\left(\frac{T_{\beta }}{T_{\alpha }}\right)=0$ , for which we want to calculate the extremum time scale τ_*p*_. Therefore, the peak relaxation time τ_*p*_, can be calculated as depicted in eq 12:

$$\ln{\left(\tau_{p}\right)}_{\pm }=\ln\left(\tau_{0,\alpha }\right)\pm \sqrt{D\left[\ln\left(\frac{\tau_{0,\alpha }}{\tau_{0,\beta }}\right)\right]}$$ 12

In order for the above equation to hold, it is necessary that *ln*(τ_0, β_) < *ln* (τ_0, α_). The minimum extremum *ln*(τ_*p*_)_−_ occurs at time scales faster than τ_0, α_, i.e., equal to $\ln\left(\tau_{0,\alpha }\right)-\sqrt{D\left[\ln\left(\frac{\tau_{0,\alpha }}{\tau_{0,\beta }}\right)\right]}$ , therefore it is outside the experimental time scales. On the other hand, the maximum extremum $\ln{\left(\tau_{p}\right)}_{+}∼\ln\left(\tau_{p}\right)=\ln\left(\tau_{0,\alpha }\right)+\sqrt{D\left[\ln\left(\frac{\tau_{0,\alpha }}{\tau_{0,\beta }}\right)\right]}$ occurs at a time scale relevant to the behavior of the materials, as shown in Figure 5b. In physical terms, the τ_*p*_ value translates to the time scale at which the temperature loss peak positions of two processes, *T*_β_ and *T*_α_, are at their closest proximity, i.e., the time scale where the double-percolation scenario is at its most limited reign. Therefore, τ_*p*_ can be approximated as the time scale at which the activation energy distribution of both the α- and β- relaxations are broader. The above analysis indicates a profound insight, that the *E*_*A*_ of the β-relaxation and the *T*_*V*_ of the α-relaxation do not play a role toward τ_*p*_; instead, τ_*p*_ is determined only by the pre-exponential factors and the fragility parameter. Finally, when no extremum is observed in the investigated time scale range like in the case of the PMMA homopolymer, it is safe to assume that $\frac{d}{d\ln\left(\tau \right)}\left(\frac{T_{\beta }}{T_{\alpha }}\right)\neq 0$ which occurs when *ln*(τ_0, β_) > *ln* (τ_0, α_). When τ_0, β_ = τ_0, α_ then the above relation yields τ_*p*_ = τ_0, β_ = τ_0, α_. The calculations are provided in the Supporting Information.

### 3.5 Chemical Composition and Charge Transport

Finally, the electrical ac conductivity as a function of MMA weight concentration is presented in Figure 6 for the random copolymers. Considering the importance of dielectric polymers in capacitor applications,^69^ as well as PMMA specifically due to its properties and processability,^70^ we briefly discuss the applicability of the random copolymers through the prism of charge transport. Comparing the PTBS homopolymer to PMMA, it is evident that charge transport was restricted, thus yielding impressive lower ac conductivity (σ′_*ac*_ = ωε_0_ε″) values by over 4 orders of magnitude at 195 °C and 10^–1^ Hz, as shown in Figure 6. The random copolymers exhibit an intermediate electrical response that strongly depends upon the MMA:TBS ratio. As discussed earlier in Figure 2a, TBS monomers are bulkier than MMA, which resulted in the reduction of the overall flexibility of the polymer chains of the random copolymers, impeding the hopping of charge carriers. It should be noted that the σ′_ac_ values are presented at 195 °C, which is 53 and 73 °C higher than the corresponding *T*_g_ *s* of PTBS and PMMA, respectively. Therefore, this 20 °C difference between the *T*_g_ values of the two homopolymers highlights the importance of *T*_g_ when considering charge transport and electrical conductivity. To highlight the fact, we present the σ′_ac_ values at 195 °C and 10^–1^ Hz as a function of calorimetric *T*_g_ as an inset in Figure 6, which indeed shows an inverse relationship.

**Figure 6:**
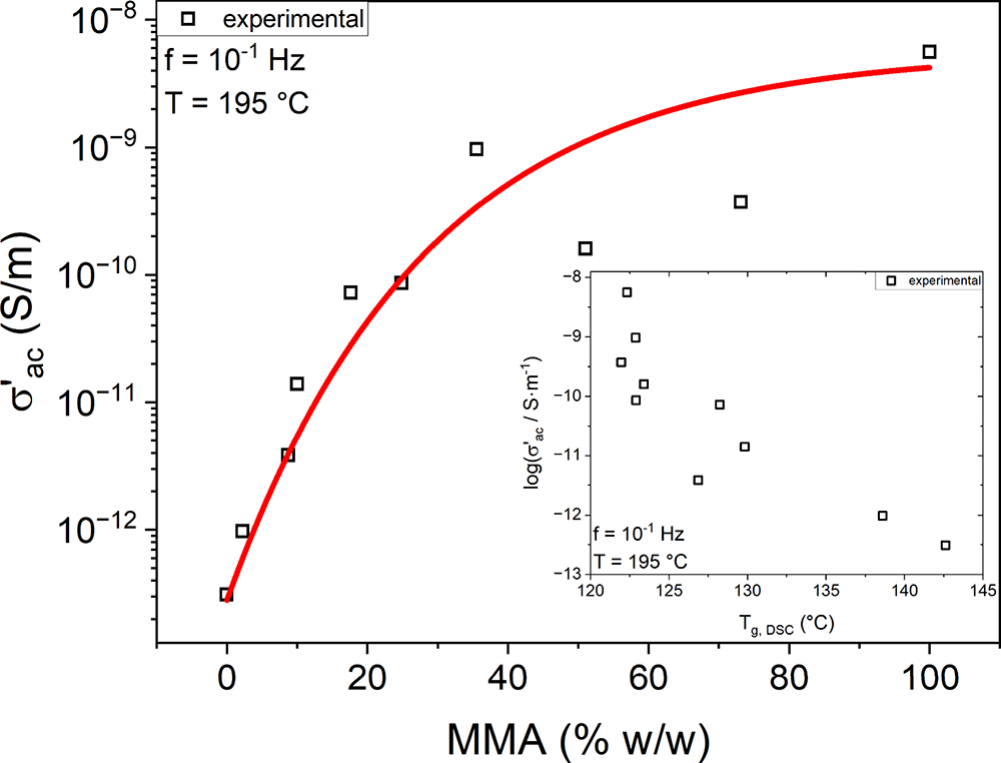
Molecular structure and charge transport. The real part of ac conductivity at 10^–1^ Hz and 195 °C against the MMA weight concentration. The red line is a guide for the eye. (Inset) Real part of ac conductivity at 10^–1^ Hz and 195 °C as a function of the calorimetric *T*_g_, where every symbol corresponds to a different P­(MMA-r-TBS) composition.

## 4 Conclusions

In the present work, we synthesized random copolymers based on PMMA and PTBS at various concentrations, to examine the resulting relaxation properties, since PMMA exhibits secondary relaxations and PTBS does not. First, we determined *T*_g_ via DSC which revealed a strong structure-dependent response and was observed to increase with the addition of the bulkier TBS units but deviated significantly from the Fox equation, highlighting the dominant role of intermolecular interactions, tacticity, free volume, and rigidity differences in governing *T*_g_.

To further examine the molecular mechanisms behind the observed trend, we investigated the α-relaxation dynamics using Broadband Dielectric Spectroscopy (BDS), revealing how monomer ratio influenced dielectric response and relaxation behavior. To interpret the calorimetric *T*_g_ results, we considered recent perspectives on how the tacticity and increasing conformational randomness, particularly in the presence of other monomers, influence the glass transition. While variations in PMMA tacticity appear to account for the observed behavior, they do not adequately explain the relaxation dynamics we observed. Our findings showed how monomer bulkiness and backbone flexibility dictate segmental dynamics. The linear relationship between fragility and *T*_g_ suggests that increased free volume enhances backbone mobility, while restricted free volume strengthens TBS’s rigid influence. This refined perspective on glass transition behavior can guide the design of polymers with optimized thermal, mechanical, and dielectric properties, facilitating advancements in coatings, soft matter physics, and high-performance materials.

Moreover, our study explored how monomer composition influences β-relaxation in copolymers of MMA and TBS. In PMMA, β-relaxation is believed to be a Johari–Goldstein mechanism driven by both backbone and side-group motions, but the incorporation of TBS disrupts this process, reducing dielectric strength and shifting relaxation peak positions. As MMA content decreases, activation energy drops significantly, indicating a transition to a relaxation mechanism likely dominated by side-group reorientation. The strong linear relationship between dielectric strength and MMA concentration further confirms that β-relaxation originates solely from MMA monomers.

At temperatures above *T*_g_, β-relaxation dynamics exhibited reduced variation between samples, while activation energy rose sharply, suggesting that free volume expansion enabled molecular mobility at greater length scales. These increased length scales suggested that backbone parts are contributing to β-relaxation, altering its behavior compared to lower temperatures below *T*_g_. While only PMMA retained the expected merger of α- and β-processes, copolymer samples with varying MMA concentrations showed similar β-relaxation trends, reinforcing the idea that TBS monomers, despite PTBS homopolymer lacking a β-process, may participate in β-relaxation at higher temperatures. These observations indicated that the high-temperature component of β-relaxation in random copolymers may correspond to a Johari–Goldstein process.

The presence of TBS in the random copolymers was found to significantly alter relaxation dynamics by slowing α-relaxation and leading to its dissociation from β-relaxation, revealing a complex interplay between mobility constraints and monomer interactions. To examine this effect, we analyzed percolation transitions between mobile and immobile regions based on a recent idea^23^ through the ratio of temperature loss peak positions (*T*_β_ */T*_α_). PMMA homopolymer exhibited a direct crossover between the two relaxations (*T*_β_ */T*_α_ = 1) whereas copolymers display distinct maxima in their *T*_β_ */T*_α_ curves, indicating a fundamental shift in relaxation behavior. By reformulating Arrhenius and VFT equations, we derived the peak relaxation time (τ_*p*_), which represents the time scale where *T*_β_ and *T*_α_ are closest. Therefore, at τ_*p*_ the double percolation scenario occurs at the minimum temperature range, and we found that it is governed by fragility and pre-exponential factors only, rather than activation energy or Vogel temperature.

This study provides a deeper understanding of how monomer ratio influences polymer relaxation dynamics, shedding light on the intricate relationship between secondary relaxations, glass transition behavior, and segmental mobility. By systematically analyzing the effect of the monomers on α- and β-relaxations, we demonstrated how molecular interactions and fragility dictate thermal and dielectric properties in random copolymers. The deviation from the Fox equation and the emergence of complex relaxation phenomena reinforce the importance of fine-tuning polymer structure to achieve tailored dynamic properties that extend beyond dielectric response. Additionally, the insights gained into relaxation decoupling and percolation transitions offer a valuable framework for understanding complicated materials by bridging fundamental polymer physics with materials properties.

## Supplementary Material



## Notes

Dielectric spectrum of PTBS above *T*_g_; relaxation map of all the samples; Arrhenius and VFT fitting parameters of the recorded relaxations of all the samples; full mathematical calculations to derive eqs 11 and 12 (PDF)
