# Supplementary material for: Segmental dynamics and local motions in disordered random copolymers
Source: Macromolecules. 2026 Apr 18;59(8):4787–99. doi: 10.1021/acs.macromol.6c00032 (PMC13130961; doi:10.1021/acs.macromol.6c00032)
Supplement: Supplementary file 1 [file ma6c00032_si_001.pdf]

Segmental dynamics and local motions in disordered random copolymers

SUPPLEMENTARY INFORMATION

Stavros X. Drakopoulos[a,*], Sundol Kim[a], Richard A. Register[a,b], Rodney D. Priestley[a,b,*]

[a] Princeton Materials Institute, Princeton University, Princeton 08540, New Jersey, United States (USA)

[b] Department of Chemical and Biological Engineering, Princeton University, Princeton 08540, New Jersey, United States (USA)

[*]E-mail: sd5541@princeton.edu; rpriestl@princeton.edu

DIELECTRIC DATA

The dielectric response of the PTBS homopolymer is presented below in Fig. S1, where, as expected, the dielectric spectrum is noisy since PTBS produces a weak dielectric signal, being a very low dielectric loss polymer. However, above its calorimetric T_g (determined by DSC), we were able to obtain its segmental relaxation which we subsequently fit with the Havriliak-Negami function. The dielectric spectrum remains noisy as the strength of the response is very weak, but we could still extract some information. In order to make sure that the peak we are measuring is in fact the α-relaxation, we cross-checked the validity of the obtained results via various approaches. First, according to our DSC measurements, the calorimetric T_g of PTBS is approximately 142.5 °C which should place the relaxation time of the sample at around 100 seconds ($f \approx 0.0016$ Hz) at that temperature. Therefore, the obtained dielectric data strongly indicate that the relaxation peak

we observe from 162 °C to 198 °C in the frequency range of 10^0 Hz to 10^6 Hz is in fact the α-relaxation and can be attributed to segmental motions. Second, the relaxation dynamics analysis conducted for the PTBS homopolymer (presented in Fig. 2c) clearly shows that the relaxation time follows the VFT temperature dependence, further evidence that it is associated with the segmental motions. Finally, a paper published by Sokolov's group in 2008 discusses the factors that affect fragility,[1] wherein the authors identified the segmental dynamics of PTBS which they measured via Dynamic Mechanical Analysis (DMA). The fragility value we calculated via Dielectric Spectroscopy (*m* ~ 129) in the present study is within the range they provide (*m* ~ 140±20).

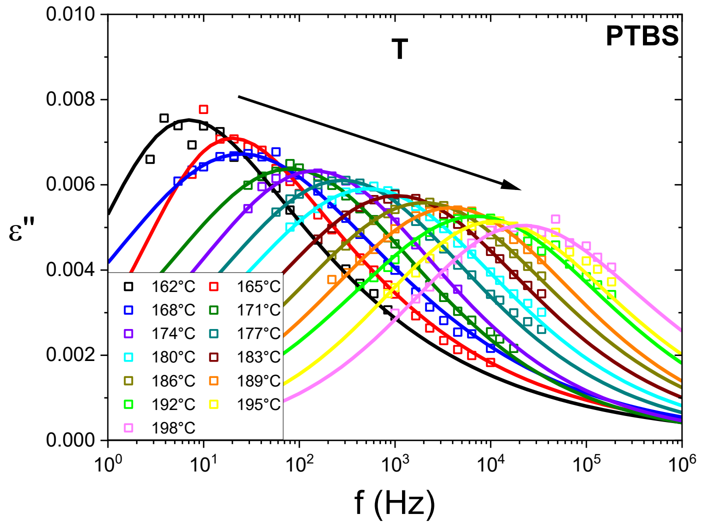

Fig. S1 | PTBS dielectric spectrum. The imaginary part of dielectric permittivity as a function of frequency at temperatures above the T_g for the PTBS homopolymer. The open symbols correspond to experimental measurements, whilst the fitting lines follow the HN function Eq. (4). PTBS possesses a very low dielectric signal that explains the noisy experimental data, and that is the reason the low frequencies are omitted from the analysis.

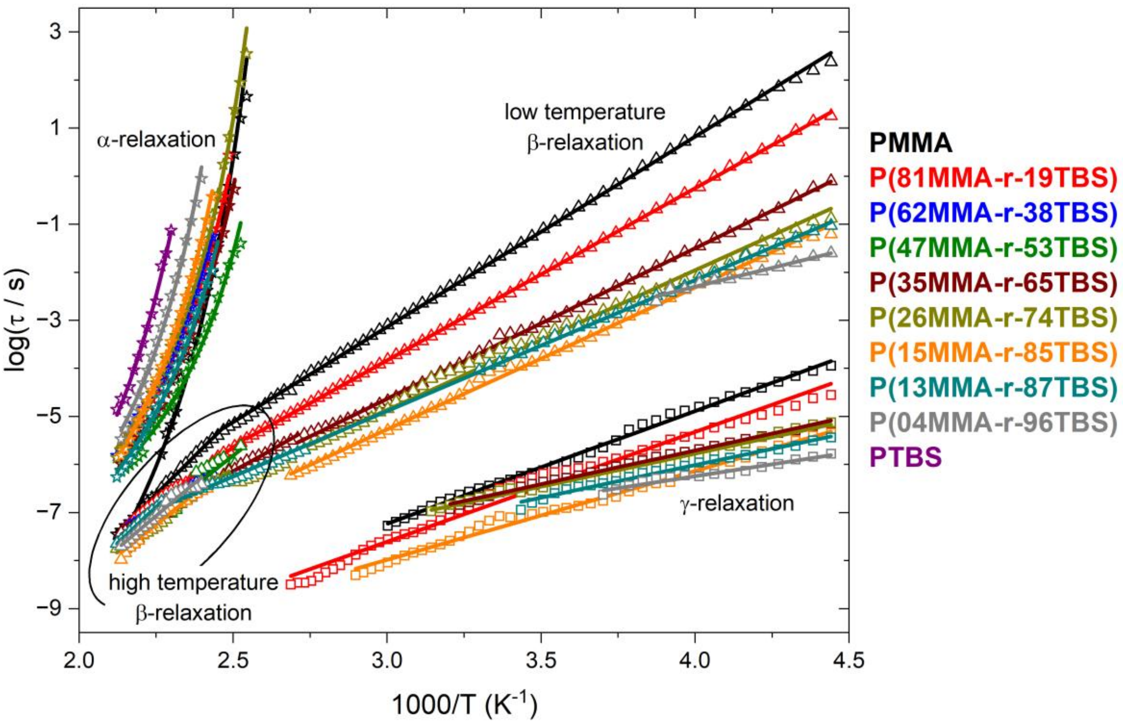

Fig. S2 | Relaxation map. The complete relaxation map of the recorded relaxations in the examined frequency and temperature ranges. The Arrhenius and VFT fitting parameters are presented below in Table S1.

Table S1 | Arrhenius and VFT fitting parameters. Fitting parameters used to fit the relaxation times of all the processes under study. With LT and HT the low-temperature and high-temperature components of the β-relaxation are designated, respectively.

Sample Name	E_A (eV)	E_A (eV)	E_A (eV)	τ_0 (s)	D	T_V (K)
	γ-relaxation	β-relaxation (LT)	β-relaxation (HT)	α-relaxation		
PMMA	0.466	0.785	1.266	1.3E-14	5.190	345.48
P(81MMA-r-19TBS)	0.453	0.71	0.926	2.6E-11	4.038	345.11
P(62MMA-r-38TBS)	–	–	0.867	2.6E-11	4.012	346.55
P(47MMA-r-53TBS)	–	–	1.001	2.4E-10	2.883	346.03
P(35MMA-r-65TBS)	0.278	0.622	0.717	3.6E-11	3.667	346.06
P(26MMA-r-74TBS)	0.272	0.577	0.919	4.7E-11	3.671	351.39
P(15MMA-r-85TBS)	0.367	0.586	1.02	5.6E-12	4.156	352.98
P(13MMA-r-87TBS)	0.248	0.54	1.196	2.7E-11	3.525	350.01
P(04MMA-r-96TBS)	0.197	0.313	1.076	5.6E-12	4.035	361.76
PTBS	–	–	–	4.7E-13	4.882	365.76

MATHEMATICAL PROCESS

Below, we show the step-by-step derivation process of:

$$\frac{T_\beta}{T_\alpha} = \left(\frac{E_A/k_B}{ln(\tau) - ln\left(\tau_{0,\beta}\right)}\right)\left(T_V + \frac{DT_V}{ln(\tau) - ln\left(\tau_{0,\alpha}\right)}\right)^{-1}$$

The first derivative with *ln(τ)* is:

$$\frac{d}{dln(\tau)}\left(\frac{T_\beta}{T_\alpha}\right) = \frac{d}{dln(\tau)}\left[\frac{\left(\frac{E_A/k_B}{ln(\tau) - ln\left(\tau_{0,\beta}\right)}\right)}{\left(T_V + \frac{DT_V}{ln(\tau) - ln\left(\tau_{0,\alpha}\right)}\right)}\right]$$

At this point we set:

$$u\big(ln(\tau)\big) = \left(\frac{E_A/k_B}{ln(\tau) - ln\left(\tau_{0,\beta}\right)}\right)$$

$$\frac{d}{dln(\tau)}\Big(u\big(ln(\tau)\big)\Big) = -\frac{E_A/k_B}{\Big(ln(\tau) - ln\left(\tau_{0,\beta}\right)\Big)^2}$$

$$v\big(ln(\tau)\big) = \left(T_V + \frac{DT_V}{ln(\tau) - ln\left(\tau_{0,\alpha}\right)}\right)$$

$$\frac{d}{dln(\tau)}\Big(v\big(ln(\tau)\big)\Big) = -\frac{DT_V}{\Big(ln(\tau) - ln\left(\tau_{0,\alpha}\right)\Big)^2}$$

We want to use the quotient rule:

$$\frac{d}{dln(\tau)}\left(\frac{T_\beta}{T_\alpha}\right) = \frac{v\big(ln(\tau)\big)\cdot\left[\frac{d}{dln(\tau)}\Big(u\big(ln(\tau)\big)\Big)\right] - u\big(ln(\tau)\big)\cdot\left[\frac{d}{dln(\tau)}\Big(v\big(ln(\tau)\big)\Big)\right]}{\Big[v\big(ln(\tau)\big)\Big]^2}$$

Therefore:

$$\frac{d}{dln(\tau)}\left(\frac{T_\beta}{T_\alpha}\right) =$$

$$= \frac{\left(T_V + \frac{DT_V}{ln(\tau) - ln(\tau_{0,\alpha})}\right) \cdot \left[-\frac{E_A/k_B}{\left(ln(\tau) - ln(\tau_{0,\beta})\right)^2}\right] - \left(\frac{E_A/k_B}{ln(\tau) - ln(\tau_{0,\beta})}\right) \cdot \left[-\frac{DT_V}{\left(ln(\tau) - ln(\tau_{0,\alpha})\right)^2}\right]}{\left[T_V + \frac{DT_V}{ln(\tau) - ln(\tau_{0,\alpha})}\right]^2}$$

We can factor E_A/k_B, T_V, and $ln(\tau) - ln(\tau_{0,\beta})$ in order to simplify the relation, as seen below:

$$\frac{d}{dln(\tau)}\left(\frac{T_\beta}{T_\alpha}\right) = \frac{\left(\frac{E_A T_V}{k_B\left(ln(\tau) - ln(\tau_{0,\beta})\right)}\right)\left(-\frac{1 + \frac{D}{ln(\tau) - ln(\tau_{0,\alpha})}}{ln(\tau) - ln(\tau_{0,\beta})} + \frac{D}{\left(ln(\tau) - ln(\tau_{0,\alpha})\right)^2}\right)}{(T_V)^2\left[1 + \frac{D}{ln(\tau) - ln(\tau_{0,\alpha})}\right]^2}$$

Finally, we can eliminate T_V from the numerator and denominator and re-order the second bracket to give the relation its final form:

$$\frac{d}{dln(\tau)}\left(\frac{T_\beta}{T_\alpha}\right) = \left(\frac{E_A}{k_B T_V\left(ln(\tau) - ln(\tau_{0,\beta})\right)}\right)\left(\frac{\frac{D}{\left(ln(\tau) - ln(\tau_{0,\alpha})\right)^2} - \frac{1 + \frac{D}{ln(\tau) - ln(\tau_{0,\alpha})}}{ln(\tau) - ln(\tau_{0,\beta})}}{\left(1 + \frac{D}{ln(\tau) - ln(\tau_{0,\alpha})}\right)^2}\right)$$

In order for $\frac{d}{dln(\tau)}\left(\frac{T_\beta}{T_\alpha}\right) = 0$ then either:

$$\frac{E_A}{k_B T_V \left(ln(\tau_p) - ln(\tau_{0,\beta})\right)} = 0$$

Which is not possible since $E_A \neq 0$, therefore:

$$\frac{\dfrac{D}{\left(ln(\tau_p) - ln(\tau_{0,\alpha})\right)^2} - \dfrac{1 + \dfrac{D}{ln(\tau_p) - ln(\tau_{0,\alpha})}}{ln(\tau_p) - ln(\tau_{0,\beta})}}{\left(1 + \dfrac{D}{ln(\tau_p) - ln(\tau_{0,\alpha})}\right)^2} = 0$$

Which translates to:

$$\frac{D}{\left(ln(\tau_p) - ln(\tau_{0,\alpha})\right)^2} - \frac{1 + \dfrac{D}{ln(\tau_p) - ln(\tau_{0,\alpha})}}{ln(\tau_p) - ln(\tau_{0,\beta})} = 0 \Leftrightarrow$$

$$\Leftrightarrow \frac{D}{\left(ln(\tau_p) - ln(\tau_{0,\alpha})\right)^2} = \frac{1 + \dfrac{D}{ln(\tau_p) - ln(\tau_{0,\alpha})}}{ln(\tau_p) - ln(\tau_{0,\beta})} \Leftrightarrow$$

$$\Leftrightarrow D\left[ln(\tau_p) - ln(\tau_{0,\beta})\right] = \left(ln(\tau_p) - ln(\tau_{0,\alpha})\right)^2 \left[1 + \frac{D}{ln(\tau_p) - ln(\tau_{0,\alpha})}\right] \Leftrightarrow$$

$$\Leftrightarrow Dln(\tau_p) - Dln(\tau_{0,\beta}) = \left(ln(\tau_p) - ln(\tau_{0,\alpha})\right)^2 + \frac{D\left(ln(\tau_p) - ln(\tau_{0,\alpha})\right)^2}{ln(\tau_p) - ln(\tau_{0,\alpha})} \Leftrightarrow$$

$$\Leftrightarrow ln(\tau_p) - Dln(\tau_{0,\beta}) = \left(ln(\tau_p) - ln(\tau_{0,\alpha})\right)^2 + \ Dln(\tau_p) - Dln(\tau_{0,\alpha}) \Leftrightarrow$$

$$\Leftrightarrow Dln\left(\frac{\tau_{0,\alpha}}{\tau_{0,\beta}}\right) = \left(ln(\tau_p) - ln(\tau_{0,\alpha})\right)^2 \Leftrightarrow$$

$$\Leftrightarrow ln(\tau_p) - ln(\tau_{0,\alpha}) = \pm\sqrt{Dln\left(\frac{\tau_{0,\alpha}}{\tau_{0,\beta}}\right)} \Leftrightarrow$$

$$\Leftrightarrow ln(\tau_p) = ln(\tau_{0,\alpha}) \pm \sqrt{Dln\left(\frac{\tau_{0,\alpha}}{\tau_{0,\beta}}\right)}$$
